# Effects of Green Manure Application on Postharvest Quality and Soil-to-Fruit Fertility Coupling in Korla Fragrant Pear (*Pyrus sinkiangensis* Yu)

**DOI:** 10.3390/biology15131070

**Published:** 2026-07-03

**Authors:** Wenyu Chen, Yongjie Liu, Minghao Sun, Jiabao Cheng, Xing Shen, Zhongping Chai

**Affiliations:** 1 College of Resources and Environment, Xinjiang Agricultural University, Urumqi 830052, China; chenwenyu666666@163.com (W.C.); sun18453303381@126.com (M.S.); chaizhongpingth@sina.com (Z.C.); 2 Korla Fragrant Pear Research Center, Bayingolin Mongol Autonomous Prefecture, Korla 841000, China; 15276268243@163.com (Y.L.); 18139663398@163.com (J.C.)

**Keywords:** Korla fragrant pear, green manure, postharvest quality, critical quality transition, soil–fruit coupling, partial-least-squares structural equation modeling (PLS–SEM)

## Abstract

Postharvest quality deterioration of Korla fragrant pear (*Pyrus sinkiangensis* Yu) severely constrains its market value, yet the regulatory role of preharvest soil management in shaping postharvest performance remains poorly understood. Although green manure is widely adopted to ameliorate orchard soil degradation, species-specific modulation of postharvest storage trajectories and the quantitative fidelity of soil-to-fruit nutrient transmission have rarely been resolved for climacteric pear species. This study investigated how green manure species modulate fruit quality at harvest and during postharvest storage life and their underlying soil–fruit linkages. Three preharvest treatments were imposed, as follows: control (CK), sweet clover (CM), and alfalfa (MX). Fruits were harvested and stored at 4 °C, with samplings at 1, 5, 10, 15, and 20 d. A critical quality transition was identified at 15 d, characterized by the concurrent peaking of soluble sugars, organic acids, vitamin C, and anthocyanins alongside an optimal sugar–acid ratio. Beyond this inflection point, CM and MX diverged markedly: CM enhanced soluble sugar accumulation, anthocyanin retention, and ester volatile production—most notably hexyl acetate, which increased over 14.4-fold—thereby generating a pronounced fruity aroma bouquet. Conversely, MX sustained higher amino acid and vitamin C levels and conferred superior late-storage stability, evidenced by a three-fold lower coefficient of variation in the sugar–acid ratio relative to CK. Partial-least-squares structural equation modeling (PLS–SEM) revealed soil fertility as the principal exploratory associative factor of fruit quality, but the fidelity of soil-to-fruit transmission was species-dependent. MX exhibited the highest observed associative strength (R^2^ = 0.971), whereas CM exhibited attenuated transmission fidelity (R^2^ = 0.777), with network analysis further indicating that CM exhibited divergent associative patterns of key soil–fruit correlations. These findings suggest that green manure identity is linked to postharvest quality through divergent soil–fruit coupling pathways: alfalfa shows nutrient transmission efficiency and stabilizes nutritional quality, whereas sweet clover promotes sugar-aroma accumulation at the cost of reduced soil–fruit conversion fidelity. Species-specific green manure selection thus offers a viable strategy for targeted modulation of postharvest traits in Korla fragrant pear.

## Simple Summary

Korla fragrant pear is a signature fruit of Xinjiang, China, prized for its aroma and sweetness. However, its quality declines rapidly after harvest, causing significant economic losses. This study investigated whether planting green manure crops—sweet clover or alfalfa—in orchards could improve the postharvest quality of the pears. We found that alfalfa green manure helped maintain higher nutritional stability during cold storage, whereas sweet clover promoted sugar accumulation and fruity aroma. Using advanced statistical models, we also showed that the type of green manure changes how soil nutrients are transferred to fruit quality. These findings suggest that growers can choose specific green manure species to target desired postharvest traits, offering a sustainable soil management strategy for pear orchards.

## 1. Introduction

Korla fragrant pear (*Pyrus sinkiangensis* Yu) is a signature product of Xinjiang’s characteristic fruit industry, renowned both domestically and internationally for its unique aroma, delicate texture, and excellent sugar–acid ratio [1,2]. It serves as a pivotal pillar industry for rural revitalization in southern Xinjiang. However, fragrant pear is a typical climacteric fruit characterized by vigorous postharvest respiratory metabolism. Postharvest loss rates of pear fruit in China are high, with quality deterioration being the primary cause [1]. Under ambient storage conditions, it rapidly exhibits peel yellowing, flesh softening, flavor deterioration, and decay, leading to a sharp decline in marketability [3,4]. Orchard soil management is a critical preharvest factor influencing postharvest fruit quality. Tree nutritional status not only determines the initial quality at harvest but also indirectly regulates postharvest senescence through its effects on cell structural integrity, antioxidant system activity, and energy metabolism levels [5]. Preharvest factors—including soil mineral nutrition, irrigation regime, tree age, and canopy management—collectively account for the majority of inter-orchard variability in pear fruit quality and storage potential [6,7]. For instance, soil potassium availability and pH have been directly linked to postharvest organic acid retention and anthocyanin stability in pear [8], while excessive preharvest nitrogen can compromise fruit firmness and storage life [9]. Green manure, as a core technology of ecological orchard cultivation, can significantly improve soil fertility and tree nutritional reserves through biological nitrogen fixation [10], organic matter (OM) return [11], and rhizosphere microecological regulation [12]. However, existing studies have predominantly focused on the effects of green manure on fruit yield and appearance quality during the growing season, while tracking studies on how green manure application influences postharvest physiological metabolism and quality maintenance through the associative effects of the “soil–tree–fruit” system remain relatively scarce.

Long-term clean-tillage management (bare-soil cultivation) in orchards has triggered soil quality degradation, including accelerated acidification, depleted organic matter, and destruction of aggregate structure [13,14,15], alongside non-point-source pollution from excessive chemical fertilization [16], ultimately compromising fruit-quality consistency and orchard sustainability [17]. Green manure, as a natural fertilizer material with soil improvement, nutrient supply, and ecological restoration functions [18], has been widely recognized as an effective approach to resolving these dilemmas [19] and promoting the green transformation of the orchard industry [20]. Fruit quality depends not only on the instantaneous state at harvest but also on its maintenance capacity during postharvest storage [21,22], encompassing appearance, nutrition, and flavor dimensions [22]. Among these, the sugar–acid ratio and vitamin C content determine nutritional quality, anthocyanin content is associated with coloration and antioxidant capacity, and volatile aroma components represent the core indicators of flavor quality [23]. There exists a quality window period, referring to a specific timeframe when comprehensive quality reaches optimal balance [24], and the formation of flavor substances is closely related to tree carbon–nitrogen metabolism and secondary metabolic pathways [25]. The soil–plant system interactive effect is key to understanding the postharvest regulatory mechanism of green manure, as soil organic matter, nutrient availability, and enzyme activity may indirectly influence postharvest storability by regulating fruit cell wall components, membrane lipid peroxidation levels, and antioxidant enzyme activity [26,27]. However, existing studies have predominantly focused on the effects of green manure on fruit yield and appearance quality during the growing season [15,20], while tracking studies on how green manure application influences postharvest physiological metabolism and quality maintenance through the “soil–tree–fruit” system remain relatively scarce [21]. Moreover, the concept of a postharvest “quality window period” has not been formally evaluated for Korla fragrant pear under different green manure regimes [24]. Consequently, the mechanistic pathways by which preharvest soil fertility is transduced into postharvest quality outcomes, and whether different green manure species create divergent soil–fruit coupling efficiencies, remain unresolved.

The effects of green manure application on the postharvest storage quality of Korla fragrant pear and its soil–fruit associative regulatory mechanism remain unclear. Based on this, the present study established three treatments—control (CK), sweet clover (*Melilotus officinalis*, CM), and alfalfa (*Medicago sativa*, MX)—and adopted a standardized storage protocol at 4 °C with a dynamic sampling strategy (0, 5, 10, 15, and 20 days). This study aims to (1) clarify the influence patterns of different green manure treatments on the dynamic changes of postharvest appearance quality, nutritional quality, and flavor substances of fragrant pear, and determine the optimal time window for quality maintenance; (2) elucidate the ameliorative effects of green manure application on rhizosphere soil fertility; and (3) analyze the association characteristics between soil and fruit quality indicators and the regulatory pathways of green manure mediating soil–fruit quality associations. Based on the identified knowledge gaps, we tested the following three hypotheses: (H1) green manure species differentially modulate postharvest quality trajectories, with alfalfa (MX) favoring nutritional stability (amino acids, vitamin C, and late-storage sugar–acid ratio consistency) and sweet clover (CM) promoting sugar accumulation and ester volatile production; (H2) a critical postharvest-quality window exists during 4 °C storage, operationally defined by the concurrent peaking of soluble sugars, organic acids, vitamin C, and anthocyanins alongside an optimal sugar–acid ratio; and (H3) the fidelity of soil-to-fruit quality transmission, quantified by PLS–SEM path coefficients (β) and explanatory power (R^2^), differs between species due to divergent rhizosphere nutrient cycling. These hypotheses were evaluated through dynamic quality profiling (days 1, 5, 10, 15, and 20), volatile metabolomics at the identified window, and structural equation modeling of paired soil–fruit datasets.

## 2. Materials and Methods

### 2.1. Experimental Materials and Site Description

The experiment was conducted at the Awaiti Farm in Bayingolin Mongol Autonomous Prefecture (86°04′ E, 41°41′ N), a core production area of Korla fragrant pear in Xinjiang, China. The experimental site is located at an altitude of 950 m, with an annual sunshine duration of 2990 h and a frost-free period of 210 d, belonging to a warm temperate continental arid climate. The soil type was irrigation-silted soil with a loam texture. Baseline soil physicochemical properties were quantified in April 2025 prior to green manure sowing. Although the experimental site had been managed under a long-term green manure rotation system, resulting in minor pre-existing differences among plots (e.g., organic matter and available phosphorus), the three treatments were arranged within a contiguous block with comparable baseline conditions, and all observed post-treatment changes were evaluated relative to these pre-planting values. The test trees were 15-year-old Korla fragrant pear, with a planting spacing of 4 m × 5 m and consistent conventional management practices.

Green manure materials included sweet clover (*Melilotus officinalis*, CM, commercial certified seeds with ≥85% germination rate, [Xinjiang Academy of Agricultural Sciences, Urumqi, China]) and alfalfa (*Medicago sativa*, MX, commercial certified seeds with ≥90% germination rate, [Xinjiang Academy of Agricultural Sciences, Urumqi, China]), which were sown in mid-April 2025 at seeding rates of 22.5 kg/ha and 15.0 kg/ha, respectively. In July 2025, at the flowering stage, the whole plants were incorporated into the soil at a depth of 15–20 cm. The green manure was sown in the inter-row spaces of the orchard, covering a total area of approximately 0.5 ha per treatment (sweet clover: ~0.5 ha; alfalfa: ~0.5 ha; control: ~0.5 ha), with a buffer zone of 10 m between treatments to minimize edge effects. The control (CK) treatment did not plant green manure; instead, naturally occurring weeds between tree rows were regularly mowed and removed.

During the 2025 growing season (April–September), the experimental site recorded the following meteorological conditions (data sources: www.nxweather.com, www.tianqi24.com, and the agrometeorological reports from the Second Division Meteorological Station, Bayingolin). In April, the mean daily temperature ranged from 12 °C to 24 °C, with an extreme maximum of 33 °C (30 April) and an extreme minimum of 5 °C (1 April); no precipitation occurred. In May, the mean temperature ranged from 19 °C to 31 °C, with extremes of 37 °C (18 May) and 12 °C (4 May); total precipitation was 3.9 mm. In June, the mean temperature ranged from 24 °C to 35 °C, with an extreme maximum of 39 °C (26 June) and an extreme minimum of 18 °C; total precipitation was 6.5 mm. In July, the mean temperature ranged from 24 °C to 36 °C, with extremes of 39 °C and 19 °C; total precipitation was 24 mm. In August, the mean temperature ranged from 20 °C to 32 °C, with extremes of 37 °C and 16 °C; total precipitation was 62.9 mm. In September, the mean temperature ranged from 13 °C to 27 °C, with extremes of 33 °C and 8 °C; total precipitation was 111.6 mm. The total precipitation during the study period (April–September) was 208.9 mm, with August and September accounting for the majority (83.9%) of rainfall, whereas April was completely dry. These climatic conditions were representative of the long-term warm temperate continental arid climate norms for the Korla region and were conducive to Korla fragrant pear development and green manure establishment.

### 2.2. Experimental Design

The field experiment was conducted using a single-factor randomized complete block design with the following three treatments: CK (control), CM (*Melilotus officinalis*), and MX *(Medicago sativa*). Throughout the experiment, all treatments received identical conventional orchard management. Drip irrigation was applied at 20-day intervals from April to September, with a total seasonal water supply of approximately 450 mm. Weed control was performed manually at 30-day intervals between tree rows. No mineral or organic fertilizers were applied during the 2025 growing season. The test trees were 15-year-old Korla fragrant pear (*Pyrus sinkiangensis* Yu) on their own roots (ungrafted), with a planting spacing of 4 m × 5 m and consistent conventional management practices.

Fruits were harvested in mid-September 2025 at commercial maturity, defined by peel color transition (green to yellow–green), soluble solids content ≥ 10.5%, and firmness of 5.5–6.5 kg/cm^2^, consistent with local industry grading standards for Korla fragrant pear [28]. For each treatment, ten fruit trees with consistent growth vigor were selected as biological replicates (*n* = 10 trees per treatment). Thirty fruits were picked from the middle part of the canopy periphery of each tree, resulting in a total of 900 fruits. All 300 fruits per treatment were pooled and stored under identical conditions. At each sampling time point, ten fruits were randomly drawn from this pooled population, with the constraint that no tree contributed more than one fruit to any single time-point analysis, thereby maintaining tree-level independence. At each storage time point, ten fruits per treatment (one fruit per tree) were randomly sampled for destructive analysis, yielding *n* = 10 biological replicates per treatment per time point. After harvest, the fruits were placed in a relatively cool area within the orchard operation room for 5 h to remove field heat. then packed into perforated plastic baskets (30 fruits per basket), transported to the laboratory, and stored in a constant-temperature light incubator at 4 ± 0.1 °C with a relative humidity of 85–90%. Samples were collected at 1, 5, 10, 15, and 20 days of storage. At each time point, ten fruits were randomly taken from each treatment. Initial quality was measured on day 1, and postharvest dynamic changes were assessed from day 5 to day 20. Fresh weight, appearance, and nutritional quality were determined immediately after sampling.

Soil samples were collected twice, as follows: before green manure incorporation (April 2025) and at fruit harvest (September 2025). Within each treatment, five trees were selected following an “S”-shaped pattern. Soil samples (0–20 cm rhizosphere soil) were collected using a soil auger at a point 10 cm inside the edge of the canopy projection. For each tree, five subsamples were taken and pooled into one composite sample. After passing through a 2 mm sieve and mixing, the soil samples were stored at 4 °C, and all analyses were completed within one week.

### 2.3. Determination Indicators and Methods

#### 2.3.1. Soil Physicochemical Properties

Soil pH was measured using a pH meter (PHS–3C, INESA Scientific Instrument, Shanghai, China). Electrical conductivity (EC) was measured using a conductivity meter (DDS–307A, INESA Scientific Instrument, Shanghai, China). Soil OM was determined by the potassium dichromate oxidation-external heating volumetric method. Total nitrogen (TN) was determined by the Kjeldahl method. Alkali-hydrolyzable nitrogen (AN) was determined by the alkali-hydrolyzed diffusion method. Available phosphorus was determined by the molybdenum–antimony resistance spectrophotometric method. Available potassium was determined by flame photometry [29].

#### 2.3.2. Fruit Quality

Appearance quality: Peel color was measured using a colorimeter (CR–400, Konica Minolta, Tokyo, Japan) at three equatorial positions per fruit, and the average was calculated. 6 as C = (a*^2^ + b*^2^)^1/2^, and hue angle (h°) was calculated as h° = arctan2(b*,a*), where arctan2 is the four-quadrant inverse tangent function that returns the correct angular value across all chromatic quadrants. The total color difference (ΔE) was calculated relative to the initial peel color at harvest (day 0) using the CIE76 formula: ΔE = [(L* − L_0_*)^2^ + (a* − a_0_*)^2^ + (b* − b_0_*)^2^]^1/2^, where L_0_*, a_0_*, and b_0_* represent the baseline color parameters measured immediately after harvest. For all fruit quality determinations, each composite sample was extracted and analyzed in triplicate (technical replicates, i.e., three repeated injections/measurements of the same extract solution) to ensure analytical precision. Biological replication was provided by the ten individual trees per treatment (*n* = 10).

Fresh weight: Single fruit fresh weight was measured using an electronic analytical balance (ME104E, Mettler Toledo, Greifensee, Switzerland). Twenty uniform-sized, pest- and disease-free fruits were selected per treatment, individually numbered, and used as fixed observation objects. Measurements were taken at 0, 5, 10, 15, and 20 d. The results are expressed as percentage weight loss relative to initial fresh weight (FW).

Soluble sugars: Soluble sugar content was determined by high-performance liquid chromatography (HPLC). Briefly, 2.0 g of pear flesh was homogenized, placed in a 50 mL centrifuge tube, and 8 mL of ultrapure water was added. The mixture was vortexed for 1 min and extracted in a water bath at 80 °C for 20 min. After cooling to room temperature, the extract was filtered through a 0.45 μm aqueous membrane filter, transferred to a 10 mL volumetric flask, and brought to volume with ultrapure water. A 1.2 mL aliquot of the sample solution was centrifuged at 10,000× *g* for 10 min, and 600 μL of the supernatant was transferred into HPLC vials. The sample was injected into an HPLC system ([Agilent 1260 Infinity II, Agilent Technologies, Santa Clara, CA, USA]) equipped with an ION–300 ion-exchange column (300 mm × 7.8 mm, 8 μm, [Transgenomic, Omaha, NE, USA]) and a UV detector ([G1314B, Agilent Technologies, Santa Clara, CA, USA]). Soluble sugars were quantified by comparison with standard substances using the external standard method, and the content of each sugar in the sample was calculated according to the standard curve. The results are expressed as mg/g fresh weight.

Organic acids: Organic acid content was determined by high-performance liquid chromatography (HPLC). Briefly, 2 g of sample was placed in a 50 mL centrifuge tube and weighed. Then, 8 mL of 0.2% metaphosphoric acid solution was added, vortexed for 1 min, and ultrasonicated at room temperature for 20 min. The mixture was filtered and brought to a final volume of 10 mL. A 1.2 mL aliquot of the sample solution was centrifuged at 10,000× *g* for 10 min, and 600 μL was transferred into two HPLC vials. The sample was injected into an HPLC system ([Agilent 1260 Infinity II, Agilent Technologies, Santa Clara, CA, USA]) equipped with an ION–300 ion-exchange column (300 mm × 7.8 mm, 8 μm, [Transgenomic, Omaha, NE, USA]) and a UV detector. The mobile phase was 50 mmol/L potassium dihydrogen phosphate solution (pH 2.5) at a flow rate of 1.0 mL/min, column temperature of 30 °C, and detection wavelength of 210 nm. Organic acid contents were quantified by comparison with standard substances using the external standard method. The results are expressed as µg/g fresh weight (FW).

Free amino acids: Amino acid content was determined by high-performance liquid chromatography with ultraviolet detection (HPLC–UV). Pear samples were crushed, and 1.0 g was weighed and brought to 25 mL with 5% trichloroacetic acid (5 g/100 mL). The mixture was homogenized, ultrasonicated for 20 min, allowed to stand for 2 h, and filtered. Then, a 1 mL aliquot of filtrate was passed through a 0.45 μm aqueous membrane filter into an HPLC vial. Internal standard norleucine was added to the sample solution, followed by pre-column derivatization with phenyl isothiocyanate (PITC). HPLC–UV analysis was performed using a Venusil–AA amino acid analysis column (100 Å, 4.6 mm × 250 mm, 5 μm, [Agela Technologies, Tianjin, China]) on an HPLC system ([Agilent 1260, Agilent Technologies, Santa Clara, CA, USA]). under the following conditions: detection wavelength 254 nm, column temperature 40 °C. Mobile phase A was sodium acetate–acetonitrile solution (pH 6.5), and mobile phase B was 80% acetonitrile. The gradient elution program was as follows: 0–0.1 min, 0% B; 0.1–14 min, 20% B; 14–33 min, 34% B; 33–41 min, 100% B; 41–49 min, 0% B. The injection volume was 2 μL, and the flow rate was 1 mL/min. Quantitative analysis of each amino acid was performed by the external standard method, and the content of each amino acid in the sample was calculated according to the standard curve. The results are expressed as µg/mL fresh weight (FW).

Anthocyanins: Total anthocyanin content was determined by the pH differential method. Fruit samples (2.0 g), with stems removed and surfaces wiped clean, were added to 20 mL of 85% hydrochloric acid–methanol solution (85:15, *v*/*v*) and extracted in the dark with shaking for 20 min. Extraction was repeated twice, the filtrates were combined and brought to 50 mL, and the filtrate was collected after filtration. A 2.0 mL aliquot of extract was brought to 10 mL with potassium chloride buffer (0.025 mol/L, pH 1.0) and sodium acetate buffer (0.4 mol/L, pH 4.5), respectively. After equilibration in the dark for 30 min, absorbance was measured at 510 nm and 700 nm using a UV-Vis spectrophotometer ([UV-2600, Shimadzu, Kyoto, Japan]). Total anthocyanin content was expressed as cyanidin-3-O-glucoside equivalents and calculated using the following formula: anthocyanin content (mg/g) = (A × MW × DF × V)/(ε × L × m), where A = (A_510_ − A_700_)pH_1.0_ − (A_510_ − A_700_)pH_4.5_, MW = 449.2, DF is the dilution factor, and ε = 26,900. Each sample was measured in triplicate. The results are expressed as mg/g fresh weight (FW).

Vitamin C: Vitamin C content was determined by titration with 2,6-dichlorophenolindophenol sodium salt (DCIP). Fruit samples (5.0 g), with stems removed and surfaces wiped clean, were added to 20 mL of 2% oxalic acid solution and ground into a homogenate under ice-bath conditions. The homogenate was transferred to a 50 mL volumetric flask, brought to volume with 2% oxalic acid solution, shaken well, and filtered. Exactly 10 mL of filtrate was pipetted into a conical flask and titrated with standardized DCIP solution until a pink color persisted for 15 s. A blank control was prepared using 2% oxalic acid solution. Each sample was measured in triplicate, and results are expressed as μg/g fresh weight.

Volatile aroma components: Volatile aroma components were determined by headspace solid-phase microextraction coupled with gas chromatography–mass spectrometry (HS–SPME–GC–MS). HS–SPME conditions: 2 g of crushed pear was placed in a 20 mL headspace vial, and 1 μL of 2-methyl-3-heptanone internal standard solution (118.2 μL/mL, [Sigma-Aldrich, St. Louis, MO, USA]) was added immediately. The vial was sealed with a polytetrafluoroethylene/butyl rubber septum. After equilibration in a 60 °C water bath for 7 min, extraction was performed using an SPME fiber (50/30 μm DVB/CAR/PDMS, [Supelco, Bellefonte, PA, USA]) for 30 min, followed by desorption at 250 °C for 7 min. High-purity helium was used as the carrier gas at a flow rate of 1 mL/min. Chromatographic conditions: analysis was performed on a GC--MS system ([Shimadzu GCMS-QP2020 NX, Shimadzu, Kyoto, Japan]) equipped with an SH–WAX capillary column (30 m × 0.25 mm × 0.25 μm, [Shimadzu, Kyoto, Japan]) was used. The column temperature program was as follows: held at 40 °C for 3 min, ramped to 100 °C at 6 °C/min, and finally ramped to 250 °C at 10 °C/min, held for 5 min. Both the detector and injector temperatures were 250 °C, with splitless injection. MS conditions: EI ion source, electron energy 70 eV, mass scan range 35–450 *m*/*z*. Ion source and interface temperatures were both 250 °C. Volatile compounds were identified using the Wiley standard mass spectral library (NIST17 and 17s) and relevant literature. In the three replicate groups of this experiment, compounds with similarity >70% were considered valid substances. If a valid substance was consistently detected in all three replicate groups, it was designated as a characteristic valid volatile compound of the sample and included in subsequent quantitative and differential analysis. If a valid substance was consistently detected in only two replicate groups, it was determined whether the compound at the same retention time in the third group was an isomer of the other two; if so, its name was modified to be consistent with the other two groups and included in subsequent analysis. The content of each volatile flavor component (μg/kg) was calculated as follows: (peak area of each component × content of internal standard)/(sample weight × peak area of internal standard). The results are expressed as µg/kg fresh weight (FW).

Sugar–acid ratio: The sugar–acid ratio was calculated as the ratio of soluble sugar content to total organic acid content, with both parameters expressed in equivalent mass units for dimensional consistency. Specifically, organic acid content (µg/g fresh weight) was converted to mg/g fresh weight (µg/g × 0.001), and the sugar–acid ratio was computed as follows:

Sugar–acid ratio = Soluble sugars (mg/g)/[Organic acids (µg/g) × 0.001]

This dimensionless ratio was calculated for each fruit sample at each storage time point to evaluate the dynamic balance between sweetness and acidity during postharvest storage.

### 2.4. Statistical Analysis

All statistical analyses were performed using R software (version [4.5.1], R Foundation for Statistical Computing, Vienna, Austria). The experimental unit was the individual tree (*n* = 10 per treatment). Although 30 fruits were harvested per tree, statistical analyses were based on tree-level means derived from one randomly selected fruit per tree per time point, avoiding pseudoreplication. Differences among treatments at each storage time point were compared by one-way analysis of variance (ANOVA), followed by Duncan’s multiple range test (*p* < 0.05). Principal component analysis (PCA) was performed using the FactoMineR package (version [2.11], R Foundation, Vienna, Austria) [30], and heatmaps were generated using the pheatmap package (version [1.0.12], R Foundation, Vienna, Austria) [31]. Figures were plotted using Origin 2023 (OriginLab, Northampton, MA, USA). To control for type I error inflation in the soil–fruit correlation networks (Figures 7 and 8), Benjamini–Hochberg false discovery rate (FDR) correction was applied across all pairwise comparisons within each treatment. Only correlations with |r| ≥ 0.8 and an FDR-adjusted q-value < 0.05 were retained as robust strong associations.

## 3. Results

### 3.1. Effects of Green Manure Application on Postharvest Nutritional Quality

#### 3.1.1. Dynamic Changes in Fruit Quality Indicators

**Figure 1:**
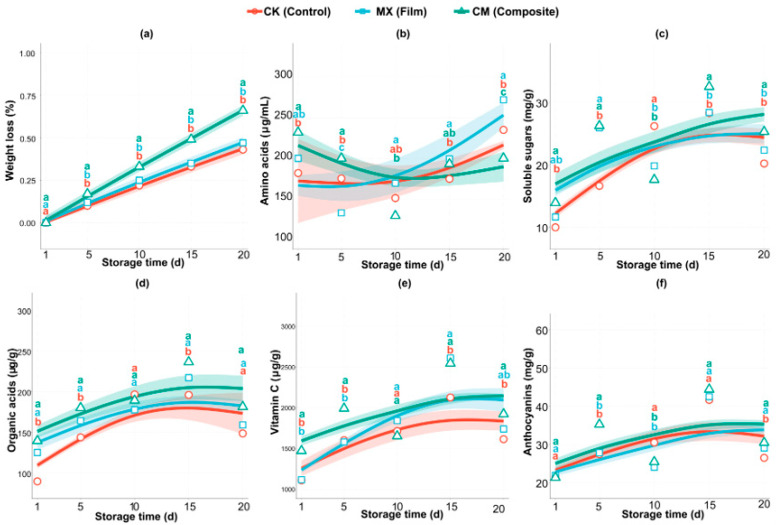
Effects of green manure treatments on fruit quality indicators of Korla fragrant pear during storage: (**a**) weight loss rate; (**b**) amino acids; (**c**) soluble sugars; (**d**) organic acids; (**e**) vitamin C; (**f**) anthocyanins. Shaded areas indicate ± SD range. Different lowercase letters indicate significant differences among treatments at the same storage time point (Duncan’s multiple range test, *p* < 0.05).

As shown in Figure 1, under 4 °C storage conditions, green manure treatments exhibited significant regulatory effects on the postharvest weight loss rate and five nutritional quality indicators of Korla fragrant pear, with each indicator showing distinct temporal dynamic variation patterns.

The weight loss rate increased progressively throughout storage across all treatments (Figure 1a). At day 20, the CM treatment exhibited the highest weight loss rate (0.66%), followed by MX (0.47%) and CK (0.43%). The CM value was significantly higher than both MX and CK at the end of storage (*p* < 0.05).

As shown in Figure 1b, amino acid content fluctuated during storage, with all treatments peaking at day 20. At this time point, MX (272.02 µg/mL) was significantly higher than CK (235.07 µg/mL) and CM (200.24 µg/mL) (*p* < 0.05).

As shown in Figure 1c, soluble sugar content showed an overall upward trend during storage, though the timing of peak accumulation differed among treatments. CM reached its maximum at day 20 (32.54 mg/g), representing a 133.4% increase over its initial value. In contrast, MX and CK peaked at day 15 (28.41 and 28.32 mg/g, respectively) and declined thereafter.

As shown in Figure 1d, organic acid content exhibited a fluctuating trend during storage. CM and MX both peaked at day 15 (236.33 and 217.13 µg/g, respectively), whereas CK peaked earlier at day 10 (196.65 µg/g). All treatments showed a decline in organic acid content by day 20.

As shown in Figure 1e, the vitamin C content increased continuously from day 1 to day 15, followed by a decline by day 20 across all treatments. At day 15, MX (2611.64 µg/g) and CM (2547.24 µg/g) were significantly higher than CK (2128.31 µg/g), representing increases of 22.7% and 19.7%, respectively.

As shown in Figure 1f, anthocyanin content increased from day 1 to day 15 and then decreased by day 20 in all treatments. At day 15, CM exhibited the highest value (44.41 mg/g), followed by MX (42.49 mg/g) and CK (41.65 mg/g).

#### 3.1.2. Dynamic Changes in Fruit Sugar–Acid Ratio

**Figure 2:**
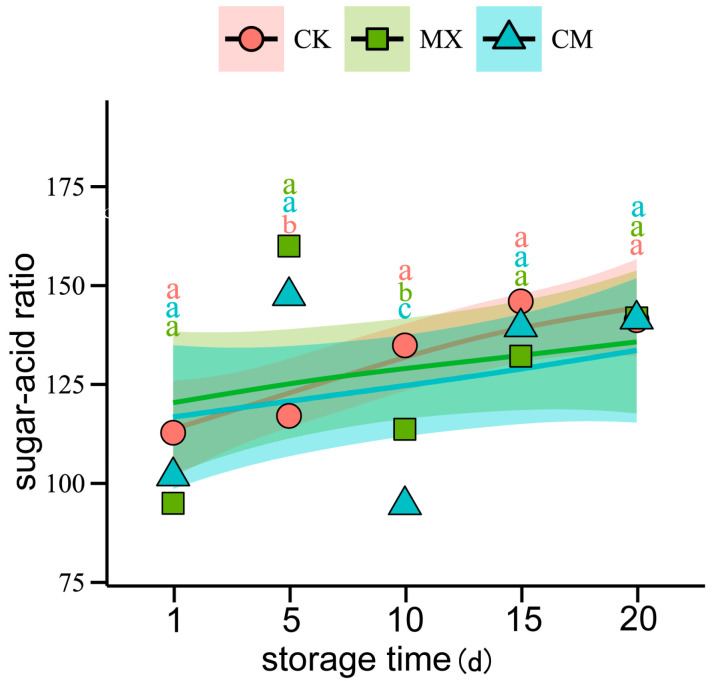
Dynamic changes in the sugar–acid ratio of Korla fragrant pear under different green manure treatments. Different lowercase letters indicate significant differences at the same storage time point (Duncan’s multiple range test, *p* < 0.05).

As shown in Figure 2, different green manure treatments exerted significant regulatory effects on the dynamic changes in the sugar–acid ratio of Korla fragrant pear during storage. At 5 days of storage, the sugar–acid ratios in the green manure treatments were significantly different from that in the CK treatment (*p* < 0.05). At 10 d, the CK treatment exhibited the highest sugar–acid ratio, which was significantly higher than those of the MX and CM treatments (*p* < 0.05), indicating that the control fruit maintained a superior flavor balance during the mid-storage period; notably, the CM treatment showed a marked decrease at this stage compared with 5 days. At 15 days and 20 days, no significant differences were observed among the three treatments (*p* > 0.05). Although the mean sugar–acid ratios converged across treatments during late storage, the coefficient of variation (CV) in the MX treatment (3.4%) was lower than that in the CK treatment (19.6%), suggesting that alfalfa green manure improved the stability of the sugar–acid ratio during the late storage period.

### 3.2. Effects of Green Manure Application on Postharvest Appearance Quality of Korla Fragrant Pear

#### Dynamic Changes in Peel Coloration

**Figure 3:**
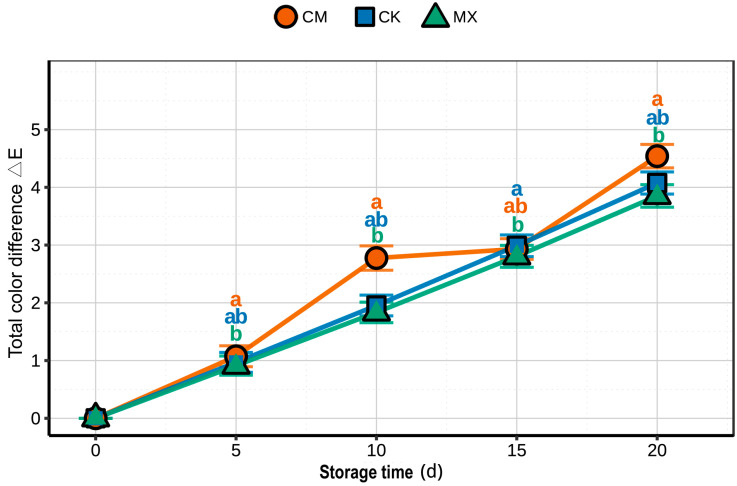
Effects of green manure treatments on postharvest peel color parameters of Korla fragrant pear. Different lowercase letters indicate significant differences at the same storage time point (Duncan’s multiple range test, *p* < 0.05).

Peel coloration is an important indicator reflecting postharvest appearance quality and senescence degree of fruits. As shown in Figure 3, under 4 °C storage conditions, the CIE Lab* color parameters of peel in Korla fragrant pear under different green manure treatments exhibited regular dynamic changes, with no significant differences in the overall trends among treatments (*p* > 0.05).

As shown in Figure 3, the total color difference (ΔE) of Korla fragrant pear peel increased gradually during cold storage across all treatments. During the early storage period (Day 1 to Day 10), ΔE values remained low (<2.0) in the control (CK), alfalfa green manure (MX), and sweet clover green manure (CM) treatments, indicating minimal deviation from the initial peel color. By Day 10, the ΔE values were approximately 1.84 (CK), 1.78 (MX), and 2.75 (CM). From Day 10 to Day 15, ΔE values increased more noticeably, reaching approximately 3.07 (CK), 2.88 (MX), and 2.98 (CM) at Day 15, suggesting the onset of chlorophyll degradation and yellowing. At Day 20, the ΔE values in CM, CK, and MX were 4.54, 4.08, and 3.85, respectively. Notably, the ΔE value in the CM treatment was significantly higher than that in the MX treatment (*p* < 0.05), whereas no significant differences were detected between CK and CM or between CK and MX (*p* > 0.05). These results indicate that sweet clover green manure accelerated peel color deviation relative to alfalfa green manure at the end of storage, although the overall temporal pattern of color change was not fundamentally altered by green manure application compared with the control.

### 3.3. Effects of Green Manure Treatments on Fruit Flavor Substances

#### 3.3.1. Effects of Green Manure Treatments on Characteristic Aroma Components of Korla Fragrant Pear During Storage

Comprehensive analysis indicated that, based on the dynamic monitoring results of the sugar–acid ratio and fruit nutritional indicators, 15 d of storage (the 4th sampling batch) was identified as the postharvest quality window period of Korla fragrant pear. This designation was operationally defined as the storage day when the maximum number of key quality indicators (soluble sugars, organic acids, vitamin C, anthocyanins, and sugar–acid ratio) simultaneously reached or approached their individual peak values within the 20-day monitoring period. At this time point, soluble sugar content reached peak values (32.54 mg/g for CM and 28.41 mg/g for MX), and the sugar–acid ratio recovered to an optimal balance (131–138). The contents of amino acids, vitamin C, and anthocyanins in the fruits were all at high levels throughout the entire storage period. During this period, the appearance quality, nutritional quality, and flavor harmony of the fruits reached the optimal balance, with the highest commercial value. Therefore, fruits at 15 d of storage were used as the material for subsequent volatile aroma component analysis and soil–fruit quality association studies, to ensure data representativeness and comparability of results. Although GC–MS quantifies volatile concentrations, we acknowledge that odor activity values (OAVs) and trained sensory panel validation were not determined in this study. Consequently, whether the observed increases in hexyl acetate and other esters exceed human detection thresholds and translate into perceptible aroma differences remains to be confirmed.

Aroma substances are the key determinants of fruit flavor quality. When soluble sugars, organic acids, anthocyanins, and vitamin C were all within the quality window period at 15 days of storage, HS–SPME–GC–MS analysis was performed on Korla fragrant pear under different green manure treatments. A total of 32 volatile aroma components were detected, including alcohols, esters, aldehydes, ketones, phenols, and alkanes.

**Figure 4:**
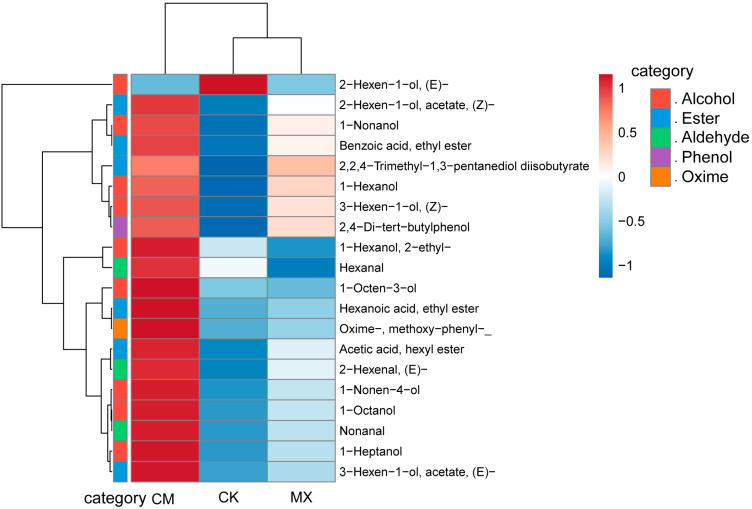
Heatmap and hierarchical cluster analysis of flavor substances in Korla fragrant pear under different green manure treatments.

As shown in Figure 4, green manure application significantly altered the postharvest volatile aroma metabolic profile of Korla fragrant pear. CM and MX clustered into one group, while CK formed a separate cluster. A total of 23 aroma components were identified, with alcohols and esters being the predominant constituents. The CM treatment exhibited the highest contents of (E)-2-hexen-1-ol and 1-Hexanol, presenting a composite aroma of green, fruity, and sweet notes; the MX treatment was enriched in esters such as ethyl hexanoate and hexyl acetate, presenting a pure and intense fruity aroma; the CK treatment displayed an overall bland flavor profile. Heatmap clustering revealed that alcohols and esters were highly expressed under green manure treatments, representing the core target substances for quality improvement. These results indicate that green manure promotes the accumulation of characteristic flavor substances by regulating aroma metabolic pathways, with CM and MX forming differentiated flavor characteristics.

#### 3.3.2. Effects of Green Manure Treatments on Characteristic Aroma Components During Storage

To intuitively demonstrate the influence of green manure treatments on characteristic aroma substances of Korla fragrant pear, six representative compounds were selected in this study based on dual criteria of content levels and flavor contribution.

**Figure 5:**
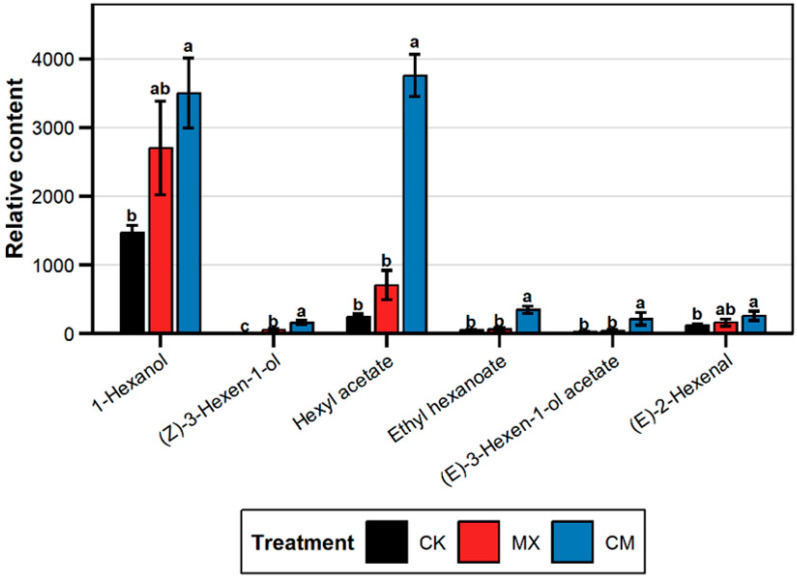
Effects of green manure treatments on characteristic aroma components of Korla fragrant pear during storage. Note: Different lowercase letters indicate significant differences at the same storage time point (Duncan’s multiple range test, *p* < 0.05).

As shown in Figure 5, the sweet clover green manure treatment (CM) significantly enhanced the contents of characteristic aroma components, exhibiting a pronounced aroma-enhancing effect. Among alcohols, the content of 1-hexanol reached 3500.95 μg/kg, representing increases of 138.3% and 29.7% compared with the control (1469.74 μg/kg) and the alfalfa green manure treatment (2699.49 μg/kg), respectively (*p* < 0.05). The content of (Z)-3-hexen-1-ol (leaf alcohol) reached 159.33 μg/kg, which was 23.3-fold and 2.9-fold higher than that of CK and MX, respectively (both *p* < 0.01). Among esters, the content of hexyl acetate was as high as 3755.83 μg/kg, representing a 14.4-fold increase compared with the control and a 4.3-fold increase compared with the alfalfa green manure treatment (*p* < 0.05). The contents of ethyl hexanoate and (E)-3-hexen-1-yl acetate were 347.15 μg/kg and 213.20 μg/kg, respectively, both significantly higher than those in the other two groups (*p* < 0.05). Among aldehydes, the content of (E)-2-hexenal reached 257.57 μg/kg, representing a 119.7% increase compared with the control (*p* < 0.05). In contrast, the alfalfa green manure treatment (MX) exhibited intermediate contents for most aroma substances between the control and the sweet clover green manure treatment, with no significant differences from the sweet clover green manure treatment only in 1-hexanol and (E)-2-hexenal (*p* > 0.05), indicating that the two green manure treatments exhibited divergent regulatory effects on fruity aroma substances (aldehydes and alcohols).

### 3.4. Effects of Green Manure Treatments on Soil Environment and Soil–Fruit Coupling Effects

#### 3.4.1. Effects of Green Manure Planting on Physicochemical Properties of Orchard Soil

**Figure 6:**
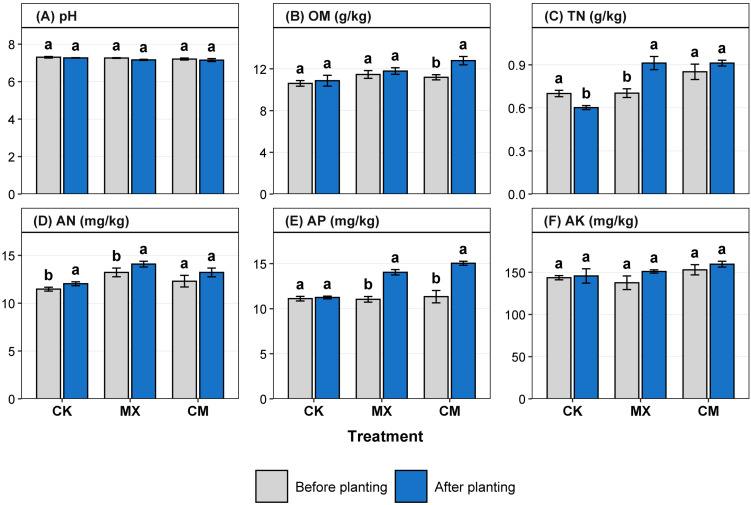
Effects of green manure planting on physicochemical properties of orchard soil: (**A**) pH; (**B**) organic matter (OM, g/kg); (**C**) total nitrogen (TN, g/kg); (**D**) alkali-hydrolyzable nitrogen (AN, mg/kg); (**E**) available phosphorus (AP, mg/kg); (**F**) available potassium (AK, mg/kg). Note: Different lowercase letters indicate significant differences at the same among treatments (Duncan’s multiple range test, *p* < 0.05).

As shown in Figure 6, green manure planting significantly altered the physicochemical properties of orchard soil. Soil pH in the MX and CM treatments decreased by 0.11 and 0.12 units, respectively, compared with pre-planting levels, whereas no significant change was observed in the CK treatment. No significant differences in soil pH were detected among treatments. whereas no significant change was observed in the CK treatment. OM content was most significantly enhanced in the CM treatment, reaching an increase of 14.3% (*p* < 0.05), while the MX treatment showed an increase of 4.6%, and no significant change was observed in the CK treatment.

Soil nitrogen status exhibited marked divergence. TN contents in the MX and CM treatments increased by 29.4% and 11.2%, respectively (*p* < 0.05), and AN showed a synchronous upward trend, with increases of 6.5% and 5.8%, respectively; nitrogen indicators in the CK treatment all decreased. Available phosphorus increased by 28.9% and 28.0% with the MX and CM treatments, respectively (*p* < 0.05), with no significant change in the CK treatment. Available potassium increased by 4.4% in the CM treatment, whereas no significant changes were observed in the MX and CK treatments, which is speculated to be related to the relatively high background potassium content in the soil of the study area.

In summary, planting both MX (alfalfa) and CM (sweet clover) green manure could effectively improve orchard soil fertility. The CM treatment exhibited the optimal enhancement effect on OM, the MX treatment made the greatest contribution to nitrogen accumulation, and both green manure treatments exerted significant promoting effects on phosphorus activation.

#### 3.4.2. Association Networks and Differential Mechanisms of Soil–Fruit Quality

**Figure 7:**
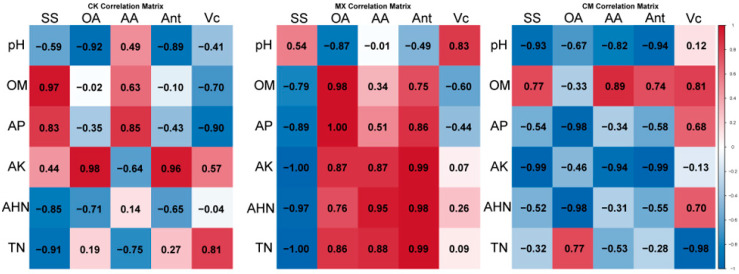
Correlation network of soil and fruit quality indicators under different treatments.

The soil–fruit quality network constructed based on Pearson correlation (Figure 7) revealed obvious differences in network topological structure among different treatment groups. The network morphology of the control group (CK) was relatively loose, with 11 strongly correlated (|r| ≥ 0.8) node pairs identified. Among these, available potassium occupied a central position, exhibiting highly positive correlations with organic acids and anthocyanins (r = 0.976 and r = 0.955, respectively); pH showed extensive negative correlations, particularly with organic acids reaching a correlation coefficient of −0.923. In contrast, the correlation of the OM node was relatively weak (r = −0.018 with organic acids).

The network structure of the MX treatment group changed significantly compared with CK. The number of strongly correlated connections in the network increased to 17, with TN and anthocyanins (r = 0.999), available potassium and anthocyanins (r = 0.998), and available phosphorus and organic acids all showing strong positive correlations. Notably, the relationship between the OM node and fruit quality indicators reversed from weak negative correlation in CK to strong positive correlation (r = 0.982). The CM treatment group exhibited different network characteristics. The proportion of negative correlations in this group increased to 64%, with directional reversal occurring in some key correlations. Specifically, the correlation coefficient between TN and vitamin C shifted from 0.809 in CK (via 0.093 in MX) to −0.997; a similar reversal occurred in the correlation between available potassium and anthocyanins.

**Figure 8:**
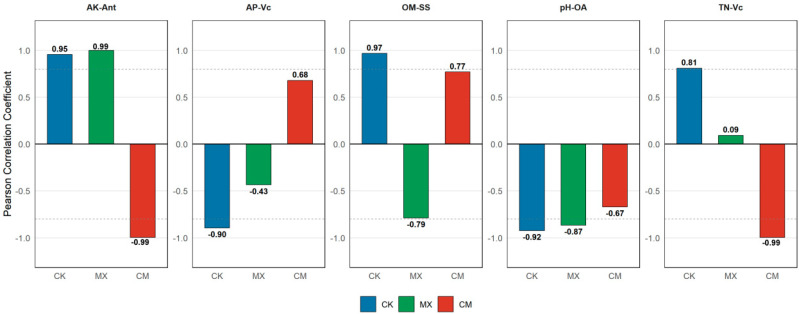
Differential comparison of key soil–fruit quality correlations among different treatments.

As shown in Figure 8, to further identify regulatory targets sensitive to management measures in the soil–fruit relationship, this study screened the five combinations with the largest coefficients of variation from all 30 indicator pairs for quantitative inter-group comparison.

The association between available potassium and anthocyanins showed treatment sensitivity similar to that of the TN–vitamin C pair. In the CK group, the two showed a strong positive correlation (r = 0.955); the MX treatment strengthened this to an extremely strong positive correlation (r = 0.999), whereas the CM treatment reversed it to a completely negative correlation (r = −0.999). The correlation between pH and organic acids remained relatively stable across the three treatment groups, with correlation coefficients of −0.923, −0.866, and −0.670, respectively, and a variation amplitude of less than 7%. The relationship between OM and soluble sugars exhibited a fluctuation pattern of “positive–negative–positive,” with correlation coefficients of 0.967, −0.788, and 0.769, respectively. The association between TN and vitamin C exhibited the most marked treatment sensitivity. The correlation coefficient attenuated from 0.809 in the CK group, through 0.093 in the MX group, to −0.997 in the CM group, with a magnitude of change reaching 1.806.

It should be noted that with *n* = 10, Pearson correlation coefficients approaching ±1.0 may reflect mathematical saturation under small-sample constraints rather than pure biological determinism. These values are presented to illustrate the topological structure of the soil–fruit association network, not to assert exact quantitative dependencies. Integrating the results of Figure 7 and Figure 8, the two green manure treatments exhibited obviously different regulatory pathways on the soil–fruit association network. The MX treatment could enhance fruit anthocyanins and organic acids. The regulatory effects of the CM treatment on specific quality indicators were divergent, requiring comprehensive consideration in combination with production objectives.

### 3.5. Correlation and Comprehensive Evaluation of Fruit Quality Indicators

**Figure 9:**
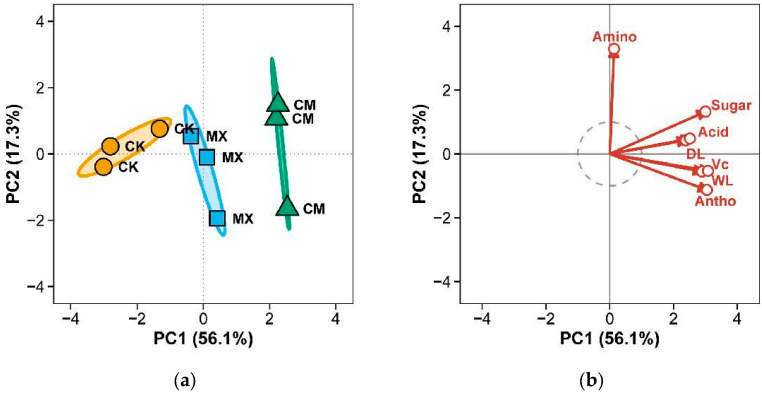
Principal component analysis of nutritional quality of Korla fragrant pear fruits under different green manure treatments: (**a**) score plot; (**b**) loading plot.

To clarify the overall effects of different green manure treatments on the nutritional quality of fragrant pear fruits, principal component analysis (PCA) was performed on seven key indicators. As shown in Figure 9, PC1 and PC2 cumulatively explained 73.4% of the variance, effectively reflecting the quality differences among samples.

As shown in Figure 9a, different treatments exhibited distinct spatial separation on the PC1–PC2 plane. CK (no-fertilizer control) was mainly distributed in the negative axis region of PC1, whereas the CM (sweet clover green manure) and MX (alfalfa green manure) treatments were oriented toward the positive axis direction of PC1, indicating that green manure application significantly altered the nutritional quality characteristics of the fruits. Among these, CM treatment samples clustered in the positive axis direction of PC2, while the MX treatment was relatively dispersed, suggesting that the sweet clover green manure treatment exhibited better consistency. Combined with the loading analysis in Figure 9b, the primary loading factors of PC1 were soluble sugars, organic acids, vitamin C, and anthocyanins, whereas PC2 primarily reflected the variation in amino acid content. The CM treatment showed positive correlations with soluble sugars, vitamin C, and anthocyanins, while the MX treatment was more strongly associated with amino acid accumulation. In summary, sweet clover green manure was conducive to enhancing the contents of soluble sugars, vitamin C, and anthocyanins in fragrant pear, whereas alfalfa green manure exerted a promoting effect on amino acid accumulation.

### 3.6. Structural Equation Model of the Relationship Between Soil Properties and Fruit Quality

#### 3.6.1. Model Specification and Sample Size Considerations

Partial least squares structural equation modeling (PLS–SEM) was conducted using SmartPLS 4 (v4.0, SmartPLS GmbH, Germany) [32] to quantify the exploratory pathway from soil fertility to fruit quality under different green manure treatments. The structural model consisted of one exogenous latent variable, soil fertility (measured by AK, AN, AP, OM, and TN), and one endogenous latent variable, fruit quality (measured by AA, Anth, OA, SS, and Vc). Separate models were estimated for the CK, MX, and CM treatments, each with *n* = 10 observations.

The sample size of 10 observations per treatment meets the absolute minimum requirement for PLS–SEM according to the “10-times rule” (i.e., 10 observations per structural path [33]); however, it falls below the more conservative threshold of 50–100 observations recommended for models with five formative or reflective indicators per construct, and below the level required for stable bootstrapped standard errors. Consequently, all parameter estimates, significance tests, and model comparisons are presented as exploratory and descriptive, and should be interpreted with appropriate caution.

#### 3.6.2. Measurement Model Assessment

The measurement model was assessed by examining outer loadings, composite reliability (CR), and average variance extracted (AVE) for the Soil Fertility and Fruit Quality constructs across the three treatments. As shown in Table 1, all indicators exhibited satisfactory loadings (> 0.70) except TN in the CK treatment (0.438), which suggests a weaker contribution to the Soil Fertility construct under conventional management.

#### 3.6.3. Discriminant Validity

Discriminant validity was assessed using the Fornell–Larcker criterion and the heterotrait–monotrait (HTMT) ratio. As shown in Table 2, the square roots of AVE (bold diagonal values) exceeded the off-diagonal Pearson correlations between latent variables for all three treatments, and all HTMT values were below the conservative threshold of 0.85.

#### 3.6.4. Structural Model Results and Interpretation

**Figure 10:**
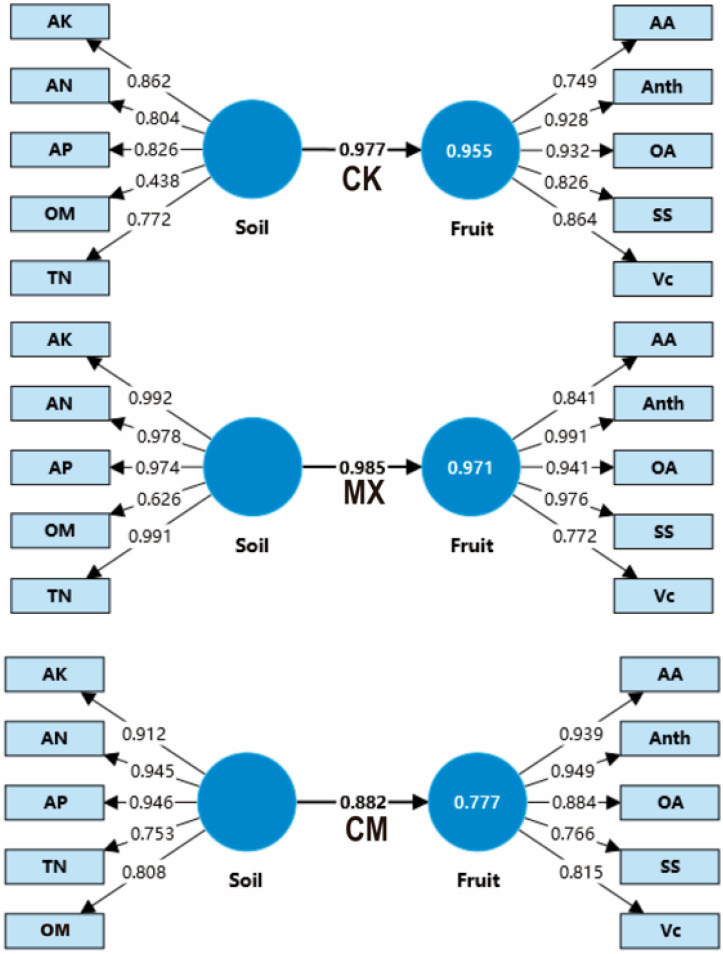
Path coefficients and factor loadings of the structural equation model for the effect of soil fertility on fruit quality under different green manure treatments.

As shown in Table 3 and Figure 10, the PLS–SEM results revealed a significant positive direct effect of Soil Fertility on Fruit Quality across all three treatments, but the transmission efficiency was species-dependent. In the control (CK), the standardized path coefficient (β) was 0.977 (*p* < 0.001), with an R^2^ of 0.955, indicating that soil nutrient status explained 95.5% of the variance in fruit quality. Under the alfalfa (MX) treatment, the soil-to-quality transmission was strongest (β = 0.985, *p* < 0.001; R^2^ = 0.971), with a path coefficient 0.8% higher than CK, suggesting that alfalfa enhanced nutrient transfer coherence through biological nitrogen fixation and root exudates. Conversely, the sweet clover (CM) treatment showed a markedly attenuated path coefficient (β = 0.882, *p* = 0.003; R^2^ = 0.777), representing a 9.7% reduction compared to CK, which implies that sweet clover may indirectly reduce the direct transmission fidelity of soil nutrients to fruit quality by altering the soil carbon-to-nitrogen ratio or root depth distribution.

Regarding the outer loadings of soil indicators (Table 1; Figure 10), available potassium (AK, 0.862), available phosphorus (AP, 0.826), and alkali-hydrolyzable nitrogen (AN, 0.804) consistently exhibited high loadings (>0.80) across all treatments, underscoring the dominant role of readily available nutrients in defining soil fertility. Notably, total nitrogen (TN) loaded weakly onto Soil Fertility in CK (0.438) but strongly in MX (0.991) and CM (0.753), suggesting that green manure incorporation enhanced the relevance of total nitrogen to the soil fertility construct.

For fruit quality indicators, organic acids (OA) and anthocyanins (Anth) displayed the highest and most stable loadings across all treatments (CK: 0.932/0.928; MX: 0.941/0.991; CM: 0.884/0.949), identifying them as robust proxies for the fruit quality latent variable. Under MX, the loading of amino acids (AA) increased from 0.749 (CK) to 0.841, whereas vitamin C (Vc) showed a lower loading (0.772) compared to CK (0.864), suggesting differential contributions of functional nutritional components under alfalfa green manure.

#### 3.6.5. Model Fit Assessment

It should be noted that the elevated HTMT values likely reflect the strong theoretical linkage between the Soil Fertility and Fruit Quality constructs: soil nutrients are the direct physicochemical substrate for fruit development, so the two constructs are expected to share substantial conceptual overlap. In small samples, this theoretical proximity becomes harder to separate statistically, but the elevated HTMT does not necessarily indicate model misspecification. Regarding SRMR, the poorer fit for CK and CM relative to MX may reflect greater within-treatment heterogeneity in conventional and sweet-clover management, respectively, rather than structural model inadequacy. The global model fit indices for the three treatments are summarized in Table 4.

## 4. Discussion

### 4.1. Defining the Postharvest Quality Window

Our results identify a distinct quality window for Korla fragrant pear at 15 days of cold storage, when soluble sugars, organic acids, vitamin C, and anthocyanins peak simultaneously and the sugar–acid ratio settles between 131 and 138 (dimensionless) [34,35,36,37]. This pattern fits the general metabolic trajectory of climacteric fruit: starch hydrolysis continues to feed soluble sugar accumulation after the respiratory climacteric, while organic acid catabolism proceeds more slowly, creating a transient period of optimal balance [37]. Similar temporal patterns have been reported in ‘Conference’ pear during cold storage, where soluble solids and titratable acidity reached an optimal balance at 14–16 days [6]. What deserves attention is the 5–d delay in the sugar peak for CM (20 d versus 15 d for MX and CK), which indicates that green manure type can shift the timing of postharvest carbon mobilization by altering preharvest reserve status [35,38]. This finding is consistent with a study on ‘Rocha’ pear, where preharvest nitrogen and water management significantly affected the timing of sugar accumulation during storage [9]. This moves the concept of harvest maturity beyond external color and firmness to include an internal metabolic rhythm programmed by orchard soil management [24]. For practice, knowing the quality window for each green manure treatment allows growers to match green manure choice to market destination—immediate fresh consumption versus extended distribution chains—rather than relying on a single harvest standard [24,36]. As a typical climacteric fruit, Korla fragrant pear produces autocatalytic ethylene via the 1-aminocyclopropane-1-carboxylic acid (ACC) synthase/oxidase pathway. We speculate that the simultaneous peaking of soluble sugars, organic acids, vitamin C, and anthocyanins at 15 days may coincide with the respiratory climacteric peak under 4 °C storage. Green manure may indirectly modulate postharvest ethylene by altering preharvest tree nutrition: higher N availability under MX could advance the climacteric via enhanced ACC synthase activity, while the carbon-rich rhizosphere under CM may delay senescence via improved energy metabolism. Direct measurement of ethylene evolution, ACC content, and respiration rate is required to test this hypothesis. These findings support Hypothesis 2 (a critical postharvest-quality window exists during 4 °C storage, operationally defined by the concurrent peaking of soluble sugars, organic acids, vitamin C, and anthocyanins alongside an optimal sugar-acid ratio).

### 4.2. Divergent Nutritional and Aroma Outcomes of CM and MX

Hypothesis 1 (green manure species differentially modulate postharvest quality trajectories, with alfalfa favoring nutritional stability and sweet clover promoting sugar accumulation and ester volatile production) was largely supported: CM and MX exerted markedly divergent effects, with CM enhancing soluble sugars, anthocyanins, and ester volatiles (e.g., 14.4-fold hexyl acetate increase), whereas MX sustained higher amino acids and vitamin C and conferred superior late-storage sugar–acid ratio stability (CV 3.4% vs. 19.6% for CK). The two green manures created clearly different domains of advantage. MX produced significantly higher fruit amino acid content than CM and CK, already evident on Day 1 of storage, which means the nitrogen benefit of alfalfa was transferred to fruit before harvest [39]. Alfalfa is a deep-rooted legume with strong biological nitrogen fixation; its residues decompose rapidly after incorporation and supply ample mineral nitrogen during the critical fruit development stage [10]. Nitrogen is the direct substrate for amino acid synthesis, and amino acids serve not only as protein building blocks but also as precursors for aroma volatiles and osmolytes [40]. This positive effect of leguminous green manure on fruit amino acid content has also been observed in apple orchards, where white clover intercropping increased fruit asparagine and glutamine levels by 25–30% [41,42]. This explains the superior nutritional quality under MX.

CM, in contrast, performed better in soluble sugars, characteristic ester volatiles, and anthocyanins. Sweet clover produces higher biomass and releases polyphenol-rich root exudates, traits that accelerate soil OM turnover and phosphorus mobilization [17,43]. In our study, CM increased soil OM by 14.3% and available phosphorus by 28.0%, both significantly higher than MX. Putatively, the higher available phosphorus observed under CM may have been associated with increased soluble sugar accumulation [44]. A mechanistic explanation involving PEPC activation or glycolytic flux would require direct enzymatic measurement, which was not performed in this study. while also promoting anthocyanin pigmentation through nutrient signaling [45]. Higher P availability has been linked to increased fruit sugar content in pear [46] and enhanced anthocyanin biosynthesis in apple [47]. More striking was the 14.4-fold increase in hexyl acetate under CM. Ester biosynthesis depends on acyl-CoA and alcohol precursors derived directly from sugar and lipid metabolism [48,49], and the carbon-rich rhizosphere environment under CM probably supplied more carbon skeletons for this pathway [50,51].

Weight loss dynamics also merit brief consideration. The CM treatment exhibited the highest weight loss rate (0.66%) at day 20, followed by MX (0.47%) and CK (0.43%). Although absolute values remained below 1% throughout storage—indicating that all treatments maintained acceptable turgor and surface integrity—the treatment-specific divergence suggests that green manure species may differentially affect preharvest fruit surface properties (e.g., cuticle thickness, wax deposition, or stomatal density) that influence postharvest transpiration. These differences in weight loss trajectory, while modest in magnitude, align with the divergent nutritional and aroma outcomes described above and warrant future investigation into preharvest epidermal development under different green manure regimes.

A contradiction emerges here: MX dominated in nitrogen supply yet fell behind CM in ester volatile production. The common assumption is that nitrogen promotes secondary metabolism, but our data point to a possible carbon–nitrogen trade-off. When nitrogen is abundant, carbon skeletons may be diverted preferentially into amino acid and protein synthesis, reducing the carbon flux available for volatile ester formation [52,53]. This trade-off has been proposed in ‘Bartlett’ pear, where high N fertilization suppressed ester biosynthesis while increasing free amino acids [54,55]. If operative in our system, CM’s relative nitrogen “restraint” may conserve carbon resources for ester synthesis. This interpretation remains speculative and should be tested through isotope labeling or metabolomic flux analysis.

### 4.3. Soil Fertility Improvement and Soil–Fruit Coupling Pathways

Hypothesis 3 (the fidelity of soil-to-fruit quality transmission, quantified by PLS-SEM path coefficients and explanatory power, differs between species due to divergent rhizosphere nutrient cycling) was partially supported: PLS–SEM revealed species-dependent associative patterns, with MX exhibiting the strongest soil–fruit transmission fidelity (β = 0.985, R^2^ = 0.971) and CM showing attenuated coherence (β = 0.882, R^2^ = 0.777) and an increased proportion of negative soil–fruit correlations (64%). PLS-SEM revealed substantial differences in standardized path coefficients from soil fertility to fruit quality, follows: 0.985 for MX, 0.977 for CK, and only 0.882 for CM [33]. This ranking does not follow the magnitude of soil fertility improvement—CM actually achieved larger gains in OM and available phosphorus than MX. Thus, better soil fertility does not automatically translate into better fruit quality; the associative strength depends on green manure type. Similar decoupling between soil nutrient status and crop quality has been reported in vineyards, where high soil fertility did not always produce superior wine grapes due to asynchronous nutrient release [56].

The high path coefficient for MX likely reflects close synchrony between nitrogen release and tree demand. We hypothesize that alfalfa residues, which have a relatively low C/N ratio and decompose rapidly, released nitrogen during the fruit cell expansion phase, potentially achieving a supply–demand match [57]. Direct isotope tracing would be required to validate this interpretation. This synchrony concept is well established in agroecology: legumes provide N in phase with crop demand, whereas residues with a higher C/N ratio can cause net N immobilization [57,58]. By contrast, the attenuated coefficient for CM suggests a decoupling mechanism. A C/N stoichiometric mismatch may explain this: sweet clover residues have a relatively higher C/N ratio than alfalfa, and during early decomposition, microbes compete with the tree for mineral nitrogen, creating a transient nitrogen deficit at a critical stage of fruit development [57,59]. A transient N deficit during fruit cell division has been linked to reduced sink strength and lower final fruit quality in pear [60]. Although postharvest soil tests showed increased total and alkali-hydrolyzable nitrogen under CM, much of this nitrogen may exist in organic forms with low immediate availability. Supporting this interpretation, the correlation between TN and vitamin C reversed from +0.809 in CK to −0.997 in CM, indicating that this nitrogen pool was not effectively accessed by the tree.

Network topology analysis corroborates this view. In the CK network, available potassium occupied a central hub, reflecting the dominance of chemical potassium fertilizer in conventional orchards [61]. The MX network added strong positive links between OM and fruit quality, showing that alfalfa-derived carbon successfully entered the soil–fruit signaling pathway. The CM network, however, showed a surge in negative correlations (64%) and polarity reversals in key associations, indicating that sweet clover disturbed the existing soil–fruit equilibrium, possibly through allelochemical release or microbial community restructuring [62,63]. Taken together, green manure evaluation should consider not only soil nutrient content but also the efficiency with which trees take up and convert these nutrients into fruit quality [64].

### 4.4. Practical Implications

The present findings provide directly actionable guidance for Korla fragrant pear orchard management. First, green manure selection should be matched to the target market channel. For orchards supplying distant markets or extended cold-storage chains, alfalfa (MX) is preferable because it maintained higher amino acid and vitamin C levels and conferred a threefold lower coefficient of variation in sugar–acid ratio during late storage (3.4% vs. 19.6% for CK), indicating superior nutritional stability. For orchards targeting immediate fresh consumption or premium aroma-driven markets, sweet clover (CM) is more suitable because it significantly enhanced soluble sugar accumulation (32.54 mg/g at day 20), anthocyanin retention (44.41 mg/g), and ester volatile production—notably hexyl acetate, which increased 14.4-fold—thereby generating a pronounced fruity aroma bouquet.

Second, the identification of a quality window at 15 days of cold storage offers a practical harvest-to-market timing benchmark [24]. Growers can use this 15-day interval as a standard for quality inspection, logistical scheduling, and shelf-life estimation, rather than relying solely on external color or firmness.

Third, the soil-to-fruit transmission efficiency (β = 0.985 for MX vs. 0.882 for CM) suggests that green manure evaluation should extend beyond conventional soil fertility metrics (OM, N, P, K) to include the coherence of nutrient transfer into fruit quality. The strong associative strength observed under MX likely reflects a closer synchrony between nitrogen release and tree demand [57], which orchard managers can use as a screening criterion: species that establish strong positive soil–fruit correlations are likely to deliver more predictable quality outcomes, whereas species that introduce negative correlation reversals may require complementary management to stabilize fruit quality.

### 4.5. Limitations and Future Directions

Several limitations of this study should be acknowledged. First, the present experiment represents a single-season trial (2025 growing season) with green manure incorporated in July and fruit harvested in mid-September; consequently, the observed soil fertility changes and fruit quality responses may reflect short-term physiological effects rather than long-term soil organic matter stabilization and nutrient cycling equilibrium. Multi-year trials are required to confirm the stability of these soil–fruit coupling pathways under inter-annual climatic variability. Second, the PLS–SEM analysis was exploratory in nature owing to the small sample size (*n* = 10 per treatment), HTMT values exceeding the conservative 0.85 threshold, and SRMR values above 0.08 for CK and CM; therefore, the path coefficients and R^2^ values should be interpreted as indicative associative patterns rather than definitive causal evidence. Third, direct measurements of ethylene evolution, ACC synthase and ACC oxidase activity, and trained sensory panel scores were not included, limiting mechanistic interpretation of climacteric metabolism and consumer-relevance validation of the identified quality windows. Although sensory evaluation is recognized as the ultimate criterion for consumer acceptance of pear fruit [1], it was not included in the present study due to the destructive nature of panel testing and the limited sample size required for volatile metabolomics and PLS–SEM analysis. Instead, we employed an integrated instrumental quality assessment—encompassing colorimetric parameters, HPLC–based nutritional profiling, and HS–SPME–GC–MS aroma analysis—to provide an objective and reproducible proxy for sensory perception [65]. Future studies will correlate these instrumental metrics with trained sensory panel scores to validate the consumer relevance of the identified quality windows. Future research should integrate (i) multi-year field trials with expanded sample sizes, (ii) metabolomic profiling of individual sugars, organic acids, and amino acids, (iii) ethylene biosynthesis monitoring during postharvest storage, and (iv) correlation of instrumental quality metrics with sensory evaluation to consolidate green manure recommendations for Korla fragrant pear production. Fourth, direct measurements of respiration rate, ethylene production, antioxidant enzyme activity (e.g., superoxide dismutase [SOD], catalase [CAT], and peroxidase [POD]), and membrane lipid peroxidation markers (e.g., malondialdehyde [MDA]) were not included. Fifth, the postharvest quality window was defined by the concurrent peaking of multiple physicochemical indicators rather than by a weighted optimization algorithm, composite index, or consumer sensory validation. This operational definition is inherently subjective and reflects a physiological rather than a sensory optimum. Their absence limits our ability to propose mechanistic explanations for the observed storage stability differences and should be prioritized in follow-up studies.

## 5. Conclusions

This study identifies a postharvest quality window for Korla fragrant pear at 15 d of cold storage, when soluble sugars, organic acids, vitamin C, and anthocyanins peak simultaneously and the sugar–acid ratio reaches an optimal range of 131–138 (dimensionless). First, sweet clover (CM) and alfalfa (MX) exert markedly different regulatory effects on fruit nutritional and aroma quality: CM significantly increases soluble sugar content (32.54 mg/g at 20 d), anthocyanin content (44.41 mg/g), and characteristic ester volatiles (hexyl acetate increased 14.4-fold), suggesting a composite aroma potential with green, fruity, and sweet notes, pending OAV and sensory validation; whereas MX significantly increases amino acid and vitamin C contents and maintains a lower coefficient of variation for the sugar–acid ratio during late storage (3.4% versus 19.6% for CK). It should be noted that peel coloration (ΔE) showed only marginal treatment-specific differences at late storage, and available potassium did not respond significantly to green manure application. Therefore, the benefits of green manure are primarily evident in nutritional and aroma traits rather than in appearance quality or potassium availability. Second, soil fertility and fruit quality exhibit positive associative patterns, but the strength of these associations varies with green manure species: MX shows the highest soil-to-fruit associative strength (β = 0.985, R^2^ = 0.970), while CM shows even lower efficiency (β = 0.882, R^2^ = 0.778) than CK (β = 0.977, R^2^ = 0.955). Third, green manure type is associated with divergent soil–fruit correlation patterns: MX strengthens positive correlations between organic matter and fruit quality indicators, whereas CM was associated with polarity reversals in key associations such as total nitrogen–vitamin C and available potassium–anthocyanins, with the proportion of negative correlations reaching 64%. Based on these findings, we recommend that green manure selection in pear orchards be tailored to target quality traits: alfalfa is preferable for orchards prioritizing nutritional quality and storage stability, while sweet clover is more suitable for enhancing sugar accumulation and aroma compound synthesis, albeit with lower direct soil–fruit transmission fidelity from soil nutrients to fruit quality.

## Figures and Tables

**Table 1:** Measurement model results: outer loadings, composite reliability (CR), and average variance extracted (AVE).

Latent Variable	Indicator	CK	MX	CM	CR (CK/MX/CM)	AVE (CK/MX/CM)
Soil Fertility	AK	0.862	0.992	0.912	0.865/0.966/0.943	0.572/0.852/0.768
	AN	0.804	0.978	0.945		
	AP	0.826	0.974	0.946		
	OM	0.772	0.626	0.808		
	TN	0.438	0.991	0.753		
Fruit Quality	AA	0.749	0.841	0.939	0.935/0.959/0.941	0.744/0.825/0.763
	Anth	0.928	0.991	0.949		
	OA	0.932	0.941	0.884		
	SS	0.826	0.976	0.766		
	Vc	0.864	0.772	0.815		

CR > 0.70 and AVE > 0.50 indicate satisfactory internal consistency and convergent validity. All outer loadings > 0.70 were retained, except TN in CK (0.438), which suggests a weaker contribution to the Soil Fertility construct under conventional management. AK, available potassium; AN, alkali-hydrolyzable nitrogen; AP, available phosphorus; OM, organic matter; TN, total nitrogen; AA, amino acids; Anth, anthocyanins; OA, organic acids; SS, soluble sugars; Vc, vitamin C.

**Table 2:** Discriminant validity assessed by the Fornell–Larcker criterion and HTMT ratio.

Treatment	Latent Variable	Soil Fertility	Fruit Quality	HTMT
CK	Soil Fertility	**0.756**		1.108
	Fruit Quality	0.977	**0.862**	
MX	Soil Fertility	**0.923**		1.018
	Fruit Quality	0.985	**0.908**	
CM	Soil Fertility	**0.877**		0.925
	Fruit Quality	0.882	**0.874**	

Bold diagonal values are the square roots of AVE. Off-diagonal values are Pearson correlations between latent variables. HTMT < 0.85 supports discriminant validity. All three treatments exhibited HTMT values exceeding the conservative threshold of 0.85, likely attributable to the small sample size (*n* = 10) and the strong theoretical linkage between soil fertility and fruit quality. These values should be interpreted with caution.

**Table 3:** Structural model results: path coefficients, explanatory power, effect sizes, and predictive relevance.

Path	β	SE	t	*p*	R^2^	Adjusted R^2^	f^2^	Q^2^
CK: Soil Fertility → Fruit Quality	0.977	0.215	4.538	<0.001	0.955	0.949	21.084	0.665
MX: Soil Fertility → Fruit Quality	0.985	0.005	188.762	<0.001	0.971	0.967	33.573	0.745
CM: Soil Fertility → Fruit Quality	0.882	0.3	2.94	0.003	0.777	0.749	3.491	0.477

β = standardized path coefficient; SE = standard error from bootstrapping (5000 subsamples, no sign changes); t = T statistic; *p* = two-tailed *p*-value; R^2^ = coefficient of determination; f^2^ = Cohen’s effect size (0.02 = small, 0.15 = medium, 0.35 = large); Q^2^ = Stone–Geisser predictive relevance (Q^2^ > 0 indicates predictive accuracy). Owing to the small sample size (*n* = 10), bootstrapped standard errors may be underestimated and *t*-values inflated; therefore, the *p*-values and significance decisions are approximate and exploratory.

**Table 4:** Global model fit indices.

Fit Index	CK	MX	CM	Recommended Threshold	Interpretation
SRMR	0.189	0.077	0.188	<0.08	MX: Good fit; CK/CM: Below threshold
d_ULS	1.973	0.33	1.948	<Bootstrap 95% CI	Exact fit not supported for CK/CM
d_G	n/a	n/a	n/a	<Bootstrap 95% CI	Not computable for saturated models

SRMR = standardized root mean square residual; d_ULS = unweighted least squares discrepancy; d_G = geodesic discrepancy. n/a, not applicable due to model saturation (one structural path per endogenous construct). Given the small sample size and model simplicity (one structural path), the SRMR values for CK and CM exceeded the conventional threshold of 0.08, suggesting that model fit is sensitive to sample size constraints. The MX model, however, achieved acceptable fit (SRMR = 0.077).

## Data Availability

The data presented in this study are available in the article. Raw data are available from the corresponding author upon reasonable request.

## Abbreviations

The following abbreviations are used in this manuscript:

AAs	Amino acids
AK	Available potassium
AN	Alkali-hydrolyzable nitrogen
ANOVA	Analysis of variance
Anth	Anthocyanins
AP	Available phosphorus
AVE	Average variance extracted
*β*	Standardized path coefficient
CK	Control
CM	Sweet clover (*Melilotus officinalis*)
CR	Composite reliability
CV	Coefficient of variation
DCIP	2,6-Dichlorophenolindophenol sodium salt
d_G	Geodesic discrepancy
d_ULS	Unweighted least squares discrepancy
EC	Electrical conductivity
EI	Electron ionization
f^2^	Cohen’s effect size
h°	Hue angle
HPLC	High-performance liquid chromatography
HPLC–UV	High-performance liquid chromatography with ultraviolet detection
HS–SPME–GC–MS	Headspace solid-phase microextraction coupled with gas chromatography–mass spectrometry
HTMT	Heterotrait–monotrait ratio
L*a*b*	CIE color space
*m*/*z*	Mass-to-charge ratio
MX	Alfalfa (*Medicago sativa*)
OAs	Organic acids
OM	Organic matter
PCA	Principal component analysis
PITC	Phenyl isothiocyanate
PLS–SEM	Partial-least-squares structural equation modeling
Q^2^	Stone–Geisser predictive relevance
R^2^	Coefficient of determination
SD	Standard deviation
SE	Standard error
SRMR	Standardized root mean square residual
SS	Soluble sugars
TN	Total nitrogen
Vc	Vitamin C
